# Novel insights in cough and breathing patterns of patients with idiopathic pulmonary fibrosis performing repeated 24-hour-respiratory polygraphies

**DOI:** 10.1186/s12931-017-0674-y

**Published:** 2017-11-13

**Authors:** Anke Schertel, Manuela Funke-Chambour, Thomas Geiser, Anne-Kathrin Brill

**Affiliations:** Department of Pulmonary Medicine, Inselspital, Bern University Hospital, University of Bern, Freiburgstrasse, 3010 Bern, Switzerland

**Keywords:** Idiopathic pulmonary fibrosis, Follow up, Cough, Respiratory rate, Breathing pattern, 24-h-respiratory polygraphy

## Abstract

**Background:**

The main symptoms of patients with idiopathic pulmonary fibrosis (IPF) are cough and dyspnea. IPF leads to a restrictive lung disorder impacting daytime and nocturnal breathing patterns. In this pilot study we assessed the course of day- and nighttime respiration, oxygenation, and cough over a period of 8 months as well as differences between wakefulness and sleep in IPF patients.

**Methods:**

Repetitive 24-h respiratory polygraphies (RP) and pulmonary function tests were performed at baseline and after 3, 4, 7 and 8 months. Cough-index, oxygenation parameters (SpO2, time with SpO2 < 90%, desaturation index), respiratory rate and heart rate were assessed for differences between wakefulness and sleep. The first and the last RP were compared to identify changes of these parameters over time. Statistical analyses were performed with Wilcoxon signed rank tests.

**Results:**

Nine IPF patients (8 male, median age 67 years (IQR 60, 77) with 37 valid 24-h RPs were included. Eight patients (88.9%) received antifibrotic treatment. Cough was more prevalent during wakefulness with a median cough-index of 14.8/h (IQR 10.9, 16.8) and 1.6/h (IQR 1.3–2.8) during sleep, *p* = 0.0039. Oxygenation parameters showed no difference, while respiratory- and heart rate were significantly higher during wakefulness. Despite stable pulmonary function tests over 8 months, the initially elevated respiratory rate increased further during wakefulness (baseline RR median 25.7/min (IQR 19.8, 26.6) vs. RR median 32.2/min (IQR 26.5, 40.9) at follow-up, *p* = 0.0273). The other respiratory parameters remained stable over time.

**Conclusion:**

Cough in IPF patients is more prevalent during wakefulness than during sleep. Further studies with a larger sample size and longer a follow-up period are needed to evaluate the role of the respiratory rate during wakefulness as a potential clinical follow up parameter in IPF.

**Electronic supplementary material:**

The online version of this article (10.1186/s12931-017-0674-y) contains supplementary material, which is available to authorized users.

## Introduction

Idiopathic pulmonary fibrosis (IPF) is a rare but devastating, chronic progressive interstitial lung disease of unidentified etiology. IPF usually manifests with dyspnea and/or dry cough and leads to a restrictive lung disorder, altered gas exchange and subsequent modifications in the breathing pattern [1–3].

Currently, the forced vital capacity (FVC) serves as the main follow up parameter to assess disease course, with a decline in FVC of ≥ 10% over 6 months being associated with worse survival [4]. However, in the era of available antifibrotic treatments this parameter might not be sensitive enough anymore to early identify further disease progression [5].

24-h-respiratory polygraphies (RP) with audio tracks provide additional information on cough, and breathing patterns and might also allow conclusions on disease progression, even if FVC remains stable. In this observational pilot study we used repetitive 24-h-RPs to analyze the course of breathing patterns, oxygenation and cough in IPF patients over a period of 8 months. Additionally, we aimed to identify differences in these parameters between wakefulness and sleep.

## Methods

### Participants and measurements

For this study, data was obtained from consecutive IPF patients who were seen in the in the Department of Pulmonary Medicine of the University Hospital Bern, Switzerland, and included in a clinical study between May 2014 and November 2016. The study was approved by the Cantonal ethics committee Bern (REC-No: KEK 002/14). All participants provided written informed consent. Inclusion criteria were an age of >18 years, the diagnosis of IPF with histological or radiological confirmed UIP pattern based on the current ATS/ERS guidelines [1, 6], and a subjectively disabling cough. Patients suffering from combined emphysema and fibrosis were excluded. As part of the study protocol each participant underwent repetitive ambulatory 24-h RPs (NOX T3, Nox Medical, Höfðatorg, Reykjavík, Iceland), standard pulmonary function tests (PFT), six-minute-walk-test (6MWT) and St. George Respiratory Questionnaire (SGRQ) at baseline and 3, 4, 7 and 8 months.

For the 24-h RPs patients were instructed to continue their normal daily routine during the recording period and to keep an activity log. Established long-term oxygen therapies were not interrupted for the study. Scoring of the RPs was carried out by a blinded investigator according to the AASM criteria [7] and the following definitions: lacking EEG-conduction in RP, the RP was manually divided into periods of assumed wakefulness and sleep with sleep being defined as periods without an upright position, no or minimal activity, audible snoring, no other sounds on the audio track and no other activity on the patients log. Cough was scored analogous to Kelsall et al. [8]. Additionally, single cough events were defined as one, or more cough sounds within less than 4 seconds and no more than 2 seconds between the single coughs, cough attacks were defined identically, but with a duration of more than 4 seconds. Furthermore, we scored sounds of throat clearing. Each of the different sounds was verified acoustically. Cough-index was calculated from the sum of all the cough sounds mentioned above. Sleep disordered breathing was defined according to the current AASM guidelines. An apnea-hypopnea index (AHI) <5/h was considered as no SDB, an AHI between ≥5/h and <15/h was classified as mild, an AHI between ≥15/h and <30/h as moderate and an AHI ≥30/h as severe OSA, respectively. Mean SpO2 (%), minimal SpO2 (%), time in hypoxia [time with SpO2 < 90% (min)], oxygen desaturation index (ODI), BNP, respiratory and heart rate were measured over 24 h and then calculated for the defined periods of wakefulness and sleep.

Based on our findings concerning the respiratory rate (RR) a post-hoc analyses was done to compare the median overall RR awake of all RPs and FVC values between the patients that were alive and those, that were deceased on March 15th, 2017.

### Statistical analyses

Statistical analyses and graphs were performed using GraphPad Prism 7.0 (GraphPad Software Inc., La Jolla, CA, USA). Distribution of data was tested with Kolmogorov-Smirnov-test. Comparisons of the parameters between sleep and wakefulness were performed with paired Wilcoxon signed rank tests from the means of the repetitive measurements of each participant. Over time, parameters were compared with paired Wilcoxon tests between first and last available RP. Data are presented as median followed by interquartile range (IQR 25, 75). Significance level was set at 0.05.

## Results

### Study participants

Overall, nine IPF patients, of whom eight received antifibrotic treatment were studied. The median treatment duration with antifibrotics at baseline was 7 months (IQR 3.5, 10.8). The median GAP-Score at baseline was 4.0 points (IQR 3.0, 5.0), corresponding to GAP-Stage 2 [9]. At the time of data collection all participants were in a clinically stable state. A summary of the baseline characteristics and lung function data are shown in Table 1. Out of the planned 45 24-h RPs 37 RPs were analyzed (*n* = 6 technical failure, *n* = 2 refused by patients).

**Table 1:** Baseline characteristics of the IPF patients

Characteristic	value
Number of participants (n)	9
Age (years)	67 (IQR 60, 77)
Male gender total [n, (%)]	8 (88.8%)
IPF duration at time of first RP (months)	9.0 (IQR 8, 28)
BMI (kg/m^2^)	24.9 (IQR 24.0, 29.8)
Current smoker [n, (%)]	0 (0%)
Former smoker [n, (%)]	8 (88.9%)
Never smoker [n, (%)]	1 (11.1%)
Pack years (years)	20 (IQR 9.3, 30)
FEV1/FVC (% predicted)	88 (IQR 86, 90)
FVC (L)	2.59 (IQR 2.2, 3.1)
FVC (% predicted)	58 (IQR 56, 77)
DLCO corr (% predicted)	41 (IQR 35, 43)
GAP-Score (points)	4.0 (IQR 3, 5)
Presence of OSA with AHI > 5/h [n, (%)]	6 (75.0%)
Presence of OSA with AHI > 15/h [n, (%)]	2 (22.2%)
CPAP treatment (n)	1 (11.1%)
Antifibrotic treatment [n, (%)]	8 (88.9%)
Pirfenidone [n, (%)]	5 (62.5%)
Nintedanib [n, (%)]	3 (37.5%)
Duration of antifibrotic treatment (months)	7 (IQR 3.5, 10.8)
Long term oxygen therapy [n, (%)]	3 (33.3%)

Values are given as median with interquartile range or absolute number n with percentage in parenthesis

*BMI* body mass index, FEV1/FVC Tiffeneau ratio, *FVC* forced vital capacity, DLCO corr: diffusion capacity for carbon monoxide, corrected for hemoglobin level, *OSA* obstructive sleep apnea

### Wakefulness and sleep

The median cough-index over 24 h was high with 9.55/h (median absolute cough count 229 per 24 h). Cough was significantly more prevalent during wakefulness (median overall cough-index awake 14.8/h (IQR 10.9, 16.8) than asleep (median overall cough-index asleep 1.6/h (IQR 1.32, 2.75); *p* = 0.0039). This was also found in the RPs at baseline, after 8 months and for the overall cough count and cough index across all valid RPs. RR and heart rate were more elevated during wakefulness compared with sleep, while oxygenation showed no significant difference. Results of oxygenation during wakefulness and sleep are displayed in Additional file 1: Table S1.

### Course of the respiratory and clinical parameters over time

**Fig. 1:**
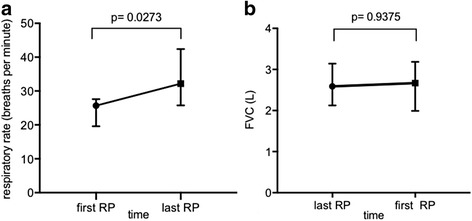
Course of the awake respiratory rate in the respiratory polygraphies (RP) and forced vital capacity (FVC) over 8 months in all nine patients

Over time the median awake RR increased significantly from 25.7/min (IQR 19.8, 26.6) to 32.2/min (IQR 26.5, 40.9), *p* = 0.0273 (Fig. 1) and remained stable during sleep (median RR asleep at baseline 20.1/min (IQR 18.9, 21.3); follow up median RR asleep 20.2/min (IQR 18.3, 23.4), *p* = 0.2109). PFT, 6MWT, oxygenation parameters, BNP and the SGRQ score remained stable. These results are summarized in Additional file 1: Table S2.

### Respiratory rate and FVC – Comparison between survivors and non-survivors

Patients were followed up for survival from study inclusion until March 15th 2017 (median follow-up time 13 months (IQR 11, 23). Five patients survived (55.6%). The median overall RR awake of all RPs was significantly higher in the non-survivors [33.1/min (IQR 24.4, 40.3)] than in the survivors [25.0/min (IQR 20.4, 30.4)], *p* = 0.0335 (Additional file 1: Figure S1 in the) while the median FVC was significantly lower in the non-surviving patients (2.1 L (IQR 1.9, 3.0) / 52% predicted (IQR 50.3, 74.0) compared to the survivors (FVC 2.8 L (IQR 2.7, 3.2) / 70% predicted (IQR 57.3, 79.8), *p* = 0.0035 / *p* = 0.0077.

## Discussion

The most important findings of this study are a significantly higher cough-index during wakefulness in IPF patients and an increase in the awake RR over time despite stable oxygenation parameters and pulmonary function tests.

Cough is frequent in IPF and the cough indices in IPF patients subjectively complaining about cough in our study are in line with the data of Key et al. who also found a median daytime cough-index of 14.6/h and significantly less coughing at night with 1.9/h [10]. The exact mechanisms by which cough is caused in IPF is not entirely known, but the pronunciation of cough during wakefulness indicates a possible mechanical influence of speech and/or physical activity and potentially increased cough reflexes that may then be diminished during sleep [10, 11].

We assume that the differences between wakefulness and sleep seen in oxygenation and heart rate with increases in heart rate and slightly worse oxygenation can be attributed to an increase in physical activity during wakefulness.

In our observational study, as an interesting finding RR during wakefulness showed a progressive and significant increase while all oxygenation parameters and pulmonary function tests, even FVC – to date the most powerful predictor of disease progression in IPF - remained stable over the study period. The overall elevated RRs are in line with former physiological studies that showed a higher resting minute ventilation in patients with IPF compared to healthy controls. This increase in resting minute ventilation was achieved by a significant increase in RR with more severe disease, while the tidal volume decreased progressively [2, 3] and FVC being inversely correlated with the RR and directly with the tidal volume [3]. One more recent laboratory study found a different breathing pattern with an increase in minute ventilation in IPF patients caused by an increase in tidal volume in daytime measurements at rest, while the RR remained stable [12]. The current study was performed in a real-life scenario with the patients being able to perform their usual daily routine while being monitored by RP at home. We could thus avoid a bias, as a study setting will inevitably influence the breathing pattern due to explicit direction of the patients’ concentration on respiration.

Until now different predictors of prognosis in IPF have been identified, including demographical, clinical, physiological and radiological aspects as well as the impact of certain comorbidities as pulmonary hypertension, emphysema, gastroesophageal reflux and lung cancer [13, 14]. GAP-Score has been developed as a model for predicting 1-year-mortality [9]. The observed progressive increase in RR during wakefulness in this study raises the question, if RR could serve as an additional and maybe more reliable follow-up-parameter and predictor for mortality in IPF. In the acute setting the RR is a valuable vital sign, as baseline tachypnea or an increasing RR during hospitalization are well-established predictors of critical illness or potentially upcoming life-threatening events as acute respiratory failure or even cardiopulmonary arrest [15, 16]. Thus, RR is part of several risk assessment scores for different medical conditions, i.e. the CURB-65 for community acquired pneumonia [17] or the Pulmonary Embolism Severity Index (PESI) [18]. In COPD patients a temporary increase in RR is also one of the earliest signs of an exacerbation and could possibly be helpful in early detection of exacerbations [19]. The baseline RR at rest or during exercise has not been found to have a prognostic value for IPF in the study by King et al. [20], but the course of RR in IPF has not been reported so far. Monitoring RR in IPF-patients might provide valuable additional information on acute disease progression and over a longer period of time. The fact, that the RR in the non-survivors was significantly higher might be an expression of a more severe restrictive lung defect and indicates, that a rise in RR could be predictive of disease progression, although this cannot be proven by means of this small pilot study.

Although our study has included only nine patients, the number of available RPs strengthens our observations. A limitation is the lack of a control group with healthy subjects or patients with other lung diseases to evaluate disease specific development of the parameters in more detail. The 24-h-RP provides information over a whole day. Thus, physical activity will influence the RR. As people were instructed to continue their normal daily routine, this could be considered as a systematical error, not influencing the course of RR. A major limitation is the use of oxygen by some patients during the RPs that is falsifying the oxygenation parameters and the results of exercise-testing. But for ethical concerns it would not have been appropriate to withdraw oxygen for 24 h in a hypoxemic and symptomatic patient. Besides having studied a population with more severe lung functional limitations and less sleep apnea this might also explain in part why in our patients the sleep desaturations did not exceed that of normal daytime activity, which has been reported before [21]. Nevertheless, despite the application of supplemental oxygen, the significant increase in RR is still detectable.

Although antifibrotic drugs reduce the decline of FVC in IPF-patients the mortality remains high. Additional parameters are needed to monitor disease progression and predict mortality [5]. RR during wakefulness might be a potential follow-up parameter, but further studies with a larger sample size and longer follow-up periods are needed to confirm our findings and test their clinical implication.

## Additional file

Additional file 1: Table S1. Respiratory parameters and cough during wakefulness and sleep. **Table S2.** Respiratory parameters, exercise tests and quality of life over time. **Figure S1.** Comparison of respiratory rate and FVC between survivors and non-survivors (DOCX 49 kb)

## Abbreviations

6MWT: Six-minute-walk-test
AHI: Apnea-hypopnea-index
AI: Apnea-index
Bpm: Beats per minute
DLCOcorr: Diffusion capacity of carbon monoxide corrected for hemoglobin level
EEG: Electroencephalography
FEV1: Forced expiratory volume in one second
FVC: Forced vital capacity
HI: Hypopnea-index
IPF: Idiopathic pulmonary fibrosis
ODI: Oxygen desaturation index (>3%)
PFT: Pulmonary function test
RP: Respiratory polygraphy
RR: Respiratory rate
SGRQ: St. George Respiratory Questionnaire
SpO2: Oxygen saturation measured by pulse oximetry
TLC: Total lung capacity
