# Supplementary material for: Novel insights in cough and breathing patterns of patients with idiopathic pulmonary fibrosis performing repeated 24-hour-respiratory polygraphies
Source: Respir Res. 2017 Nov 13;18:190. doi: 10.1186/s12931-017-0674-y (PMC5683431; doi:10.1186/s12931-017-0674-y)
Supplement: Additional file 1: Table S1. — Respiratory parameters and cough during wakefulness and sleep. Table S2. Respiratory parameters, exercise tests and quality of life over time. Figure S1. Comparison of respiratory rate and FVC between survivors and non-survivors (DOCX 49 kb) [file 12931_2017_674_MOESM1_ESM.docx]

**Additional file 1**

**eTable 1: Respiratory parameters and cough during wakefulness and sleep**

| **Parameter** | **awake** | **asleep** | **p-value** |
| --- | --- | --- | --- |
| *Oxygenation* |  |  |  |
| Mean SpO2 (%) | 90.6 (IQR 88.1, 91.5) | 90.3 (IQR 89.9, 90.4) | >0.9999 |
| Minimal SpO2 (%) | 66.8 (IQR 66.5, 70.0) | 79.5 (IQR 76.0, 82.5) | 0.0039 |
| SpO2 <90% (min) | 232.5 (IQR 137.0, 335.3) | 187.3 (IQR 106.4, 238.3) | 0.1641 |
| ODI (desaturations/h) | 12.8 (IQR 12.0, 21.3) | 7.7 (IQR 6.6, 14.8) | 0.0977 |
| Respiratory rate (breaths/min) | 28.9 (IQR 25.0, 31.0) | 20.1 (IQR 19.3, 22.6) | 0.0078 |
| Heart rate (bpm) | 81.9 (IQR 77.2, 85.5) | 64.0 (IQR 60.8, 67.9) | 0.0039 |
| *Cough* |  |  |  |
| Cough count (n) | 211 (IQR 169.5, 69.0) | 15.0 (IQR 12.8, 21.5) | 0.0039 |
| Cough-index (events/h) | 14.8 (IQR 10.9, 16.8) | 1.6 (IQR 1.32, 2.75) | 0.0039 |

**eTable 1.** The analysis was performed across all 37 valid respiratory polygraphies. Values are given as median followed by interquartile range. Abbreviations: SpO2: oxygen saturation, measured by pulsoximetry, ODI: oxygen desaturation index (>3%).

**eTable 2: Respiratory parameters, exercise tests and quality of life over time**

| **Parameter** | **baseline** | **Follow-up**  **(last available RP)** | **p- value** |
| --- | --- | --- | --- |
| *Oxygenation and sleep* |  |  |  |
| Mean SpO2 awake (%) | 90.7 (IQR 89.8, 92.4) | 90.2 (IQR 87.9, 92.3) | >0.9999 |
| Mean SpO2 asleep (%) | 89.4 (IQR 87, 90.3) | 90.8 (IQR 90, 91.6) | 0.5781 |
| Minimal SpO2 awake (%) | 69.5 (IQR 63.8, 72) | 71 (IQR 60, 73) | 0.8828 |
| Minimal SpO2 asleep (%) | 78.5 (IQR 74, 82) | 82.0 (IQR 76.0, 82) | 0.8594 |
| Time with SpO2 <90% awake (min) | 226.8 (IQR 142.3, 272) | 240.8 (IQR 37.3, 411) | 0.8438 |
| Time with SpO2 <90% asleep (min) | 262.9 (IQR 152.4, 314) | 153.2 (IQR 69, 173.5) | 0.1953 |
| ODI awake (events/h) | 11.3 (IQR 8.5, 15.3) | 17.5 (IQR 10, 22.1) | 0.1875 |
| ODI asleep (events/h) | 10.4 (IQR 5.6, 17.2) | 10 (IQR 8.3, 17.8) | >0.9999 |
| AHI (events/h) | 8.6 (IQR 5, 14.8) | 6.2 (IQR 4.9, 14.8) | >0.9999 |
| AI (events/h) | 1.0 (IQR 0.5, 1.4) | 0.4 (IQR 0.2, 1.3) | 0.4375 |
| HI (events/h) | 7.9 (IQR 2.9, 14) | 5.9 (IQR 3.6, 12.8) | 0.3125 |
| RR awake (breaths per min) | 25.7 (IQR 19.8, 26.6) | 32.2 (IQR 26.5, 40.9) | 0.0273 |
| RR asleep (breaths per min) | 20.1 (IQR 18.9, 21.3) | 20.2 (IQR 18.3, 23.4) | 0.2109 |
| Heart rate awake (bpm) | 85.5 (IQR 78.9, 90.2) | 80.7 (IQR 74.1, 84.3) | 0.0547 |
| Heart rate asleep (bpm) | 64.6 (IQR 62.6, 73.3) | 62.7 (IQR 57.2, 70.3) | 0.3828 |
| BNP (pg/ml) | 21 (IQR 10, 30) | 30 (IQR 10, 51) | 0.4375 |
| *Pulmonary function test* |  |  |  |
| TLC (L) | 4.33 (IQR 3.2, 4.4) | 4.59 (IQR 3.4, 4.9) | 0.4258 |
| TLC (% predicted) | 58 (IQR 53, 65) | 63 (IQR 56, 81) | 0.1992 |
| FEV1/FVC (%) | 88 (IQR 86, 90) | 89 (IQR 86, 90) | >0.9999 |
| FEV1 (L) | 1.9 (IQR 1.9, 2.7) | 2.03 (IQR 1.8, 2.8) | 0.9102 |
| FEV1 (% predicted) | 66 (IQR 59, 91) | 68 (IQR 57, 95) | 0.9258 |
| FVC (L) | 2.59 (IQR 2.2, 3.1) | 2.67 (IQR 2.1, 3.1) | 0.9375 |
| FVC (% predicted) | 58 (IQR 56, 77) | 59 (IQR 58, 79) | 0.6016 |
| DLCO corr (mmol/(min*kPa) | 3.5 (IQR 3.3, 4) | 3.6 (IQR 3, 4.3) | 0.6562 |
| DLCO corr (% predicted) | 40.5 (IQR 35.3, 43) | 38 (IQR 32, 51) | 0.7344 |
| *Cough* |  |  |  |
| Cough count awake (n) | 308 (IQR 210.0, 469) | 250 (IQR 217.3, 324) | 0.2188 |
| Cough count asleep (n) | 13 (IQR 6.0, 18) | 31 (IQR 13.3, 52.5) | 0.6250 |
| Cough index awake (events/h) | 19.7 (IQR 12.8, 34.2) | 16.8 (IQR 15.6, 20.6) | 0.2188 |
| Cough index asleep (events/h) | 1.8 (IQR 0.6, 2.1) | 3.45 (IQR 1.8, 5.38) | 0.5625 |
| *Exercise test* |  |  |  |
| 6MWT distance (m) | 452.5 (IQR 396.3, 505.5) | 400 (IQR 360, 500) | 0.1953 |
| 6MWT distance (% predicted) | 84 (IQR 77.8, 94.8) | 75 (IQR 59, 93) | 0.1016 |
| 6MWT baseline SpO2 (%) | 89 (IQR 88.5, 91.3) | 90 (IQR, 87, 92) | 0.8125 |
| 6MWT SpO2 minimal (%) | 79 (IQR 76.8, 83.8) | 79 (IQR 79, 83) | 0.5000 |
| *Quality of life* |  |  |  |
| SGRQ total | 52.5 (IQR 46.4, 69.2) | 60.0 (IQR 48, 73.6) | 0.2969 |
| SGRQ symptom | 59.7 (IQR 39.8, 70.4) | 59.1 (IQR 47.6, 67.4) | >0.9999 |
| SGRQ activity | 72.4 (IQR 60.4, 79.7) | 85.8 (IQR 72.3, 92.5) | 0.1250 |
| SGRQ impact | 37.7 (IQR 33.6, 65) | 45.5 (IQR 35, 70.5) | 0.3750 |

**eTable 2**. Parameters of oxygenation, sleep disordered breathing, pulmonary function test, 6-minute-walk-test and quality of life, evaluated by St. George Respiratory Questionnaire compared over an 8-months-period. Values given as median with interquartile range. Abbreviations: SpO2: oxygen saturation measured by pulsoximetry; ODI: oxygen desaturation index (>3%); AHI: apnea-hypopnea-index, AI: apnea-index, HI: hypopnea-index, RR: respiratory rate, bpm: beats per minute, TLC: total lung capacity, FEV1: forced expiratory volume in one second, FVC: forced vital capacity, DLCO corr: diffusion capacity of carbon monoxide corrected for hemoglobin level, 6MWT: six-minutes-walk-test, SGRQ: St. George Respiratory Questionnaire.

**eFigure 1 Comparison of respiratory rate and FVC between survivors and non-survivors**

**eFigure 1:** Overall median respiratory rate awake of all RPs [A] and overall FVC (L) [B] compared between the survivors and non-survivors [median RR survivors (bpm) 25.0/min (IQR 20.4, 30.4)] vs. median RR non-survivors (bpm) 33.1/min (IQR 24.4, 40.3), p= 0.0335]; [median FVC survivors (L) 2.8 (IQR 2.7, 3.2) vs. median FVC non-survivors (L) 2.1 (IQR 1.9, 3.0), p=0.0035]. Median time of follow up from study inclusion to death or alternatively March 2017 was 13 months (IQR 11, 23).
